# Automatic Segmentation of the Left Ventricle in Cardiac MRI Using Local Binary Fitting Model and Dynamic Programming Techniques

**DOI:** 10.1371/journal.pone.0114760

**Published:** 2014-12-11

**Authors:** Huaifei Hu, Zhiyong Gao, Liman Liu, Haihua Liu, Junfeng Gao, Shengzhou Xu, Wei Li, Lu Huang

**Affiliations:** 1 College of Biomedical Engineering, South-Central University for Nationalities, Wuhan, People's Republic of China; 2 College of Computer Science, South-Central University for Nationalities, Wuhan, People's Republic of China; 3 Department of Radiology of Tongji Hospital affiliated to Huazhong University of Science and Technology, Wuhan, People's Republic of China; VCU, United States of America

## Abstract

Segmentation of the left ventricle is very important to quantitatively analyze global and regional cardiac function from magnetic resonance. The aim of this study is to develop a novel algorithm for segmenting left ventricle on short-axis cardiac magnetic resonance images (MRI) to improve the performance of computer-aided diagnosis (CAD) systems. In this research, an automatic segmentation method for left ventricle is proposed on the basis of local binary fitting (LBF) model and dynamic programming techniques. The validation experiments are performed on a pool of data sets of 45 cases. For both endo- and epi-cardial contours of our results, percentage of good contours is about 93.5%, the average perpendicular distance are about 2 mm. The overlapping dice metric is about 0.91. The regression and determination coefficient between the experts and our proposed method on the LV mass is 1.038 and 0.9033, respectively; they are 1.076 and 0.9386 for ejection fraction (EF). The proposed segmentation method shows the better performance and has great potential in improving the accuracy of computer-aided diagnosis systems in cardiovascular diseases.

## Introduction

Cardiovascular disease is an important health concern due to its largest cause of death on human beings [1], [2]. The left ventricle (LV) in the short-axis orientation is particularly suitable for the assessment of stroke volume, ejection fraction, and myocardial mass, as well as regional function parameters such as wall motion and wall thickening. To perform these quantification tasks, the left ventricle needs to be segmented well.

Currently, manual segmentation of LV by an expert reader is the standard clinical practice. However, this work is a long and tedious task, with inter- and intra-observer variability. Therefore, it is attractive to develop algorithms that are accurate and require as little user interaction as possible for clinical applications [3].

MR cardiac images present a number of features that make them difficult to segment. First, it is much difficult to segment LV with left ventricular outflow tract (LVOT), since edge information is severely missing for such myocardium. Second, they may present blurred edges due to the partial volume effect (PVE) and can be affected by motion artifacts, low contrast between the myocardium and surrounding tissues complicates the segmentation further. Third, the myocardium/background contrast is lower than the myocardium/blood pool contrast. It makes the inferior and lateral sectors of the epicardial boundary very ambiguous. Finally, there is variability in the shape of the endo- and epi-cardial contours across slices and phases which make the myocardium detection much complex.

In recent years, many algorithms have been introduced to for (semi-) automated LV segmentation. They can be classified into two generic categories: (i) Deformable models, and (ii) Image-based techniques. A complete review of recent literature describing cardiac segmentation techniques is given in [4].

Deformable models include snakes [5]-[7], level set [8], [9], and their variants [10]–[12]. Deforming curves are derived iteratively according to the minimization of an energy function which consists of a data-driven term. A stochastic active contour scheme for automatic image segmentation was proposed in [13]. This method uses a parametric shape prior and integrates the region and boundary information into one generalized energy function for minimization, which is then driven by the calculus of variation under the framework of level set. The difficulty lies in the modeling which requires the prior knowledge of the heart for a better assessment of the object boundary.

Image-based techniques include thresholding [14], pixel classification [15]–[17], region-based and edge-based techniques. Lu [18] and Huang [19] adopted Otsu thresholding method [20] for segmentation of left ventricle in cardiac cine MRI. However, Otsu method is based on the histogram of objects in the image and could be biased from the optimal threshold [21]. Dynamic programming (DP) is used to detect borders of left ventricle in magnetic resonance images [22], [23]. However, the classic DP sometimes works poorly in boundary extraction due to complex edge information of the myocardium [24].

In this study, an image-based segmentation schema which combines deformable model and image-based techniques is proposed to improve the segmentation performance. In our schema, a slice by slice segmentation of the LV image starts from mid-slice to the top end slice and the bottom end slice separately. For a slice image, LBF model [25] is used to locate the blood pool first. For LVOT images, an initial endocardial contour is extracted using context information from the previous slice image; while for the images without LVOT, it is convex hull of the detected blood pool's boundary. At last, dynamic programming techniques are used to obtain the epicardial boundary. Prior information about spatial relationships between slice images is considered in this process. In other words, for all slice images, the results of current slice image are used to restrict the processing region for the next slice image, which makes the segmentation accurate and robust.

## Materials and Methods

### 1.1 Data set

In this paper, the datasets are cardiac cine MRI short axis images published by MICCAI grand challenge on the Internet (http://sourceforge.net/projects/cardiac-mr/files/). There are 45 cases containing 12 heart failure with ischemia (HF-I) cases, 12 heart failure without ischemia (HF-NI) cases, 12 hypertrophy (HYP) cases and 9 normal (N) cases. The image data were obtained during 10-15 second breath-holds with a temporal resolution of 20 cardiac phases over the heart cycle. 6 to 12 SAX images were obtained from the atrioventricular ring to the apex (thickness = 8∼10 mm, FOV = 320 mm 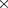 320 mm, matrix = 256 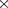 256).

### 1.2 The Framework of Segmentation Algorithm

A slice by slice LV segmentation schema is proposed which can utilize the strong temporal correlation between neighboring slices.

**Figure 1:**
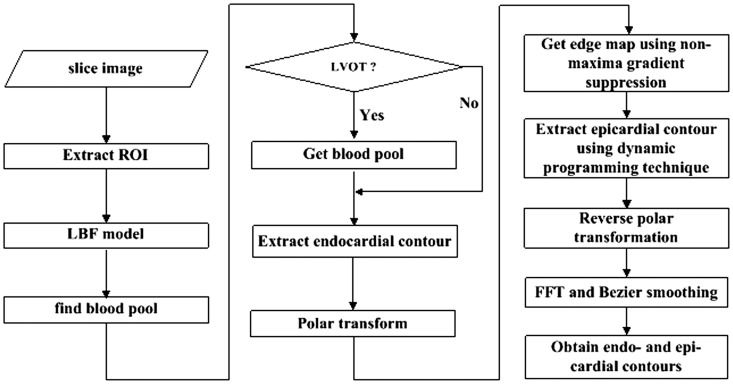
Workflow of our segmentation algorithm. LVOT: left ventricular outflow tract.

The whole procedure of one slice image segmentation consists of using a series of image processing techniques as depicted in Fig. 1. The algorithm starts from locating the region of interest (ROI) in the processing slice. Subsequently, local binary fitting model is used to find blood pool in the ROI. Then, threshold is obtained to get a refined blood pool. Two different types of slice image, i.e. with and without LVOT, are detected and corresponding techniques are processed to obtain the endocardial contour. To extract epicardial contour, the gradient image is calculated and a non-maxima gradient suppression technique is adopted to get an edge map, and region constrained dynamic programming is then employed. Details of our segmentation method are described in the following sections.

### Step 1: ROI and LV Location

We begin the segmentation from mid-slice image of carcadic MRI. For most cases, notice that the LV blood pool is located in the middle of the mid-slice image. A rectangular ROI is automatically specified centered at the mid-slice image. When segmenting a slice from mid-slice to two-ends, we use the center point of the endocardial of its previous slice as the center of current ROI.

Given ROI including the LV, there are several tissues, such as LV blood pool, myocardium, right ventricle (RV) blood pool, lung and stomach. Different tissue has different image intensity and edge information. Hence, the intensity distribution and edge information are good characters in active contour model for image segmentation. Let 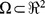 be the image domain, 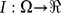 be the given ROI image, Chunming Li formulated the image segmentation problem as follows: given an image I, find a contour 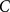 which segments the image into non-overlapping regions [25]. The proposed the energy functional as follows: 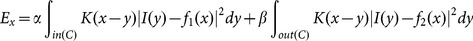
(1)


Where 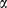 and 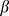 are positive constants, and 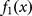 and 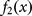 are two values that approximate image intensities of the regions inside and outside the contour 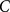 respectively. The intensities 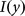 are in local region centered at the point 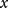, whose size can be controlled by a Gaussian kernel function 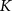, as explained below.
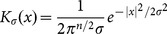
(2)


**Figure 2:**
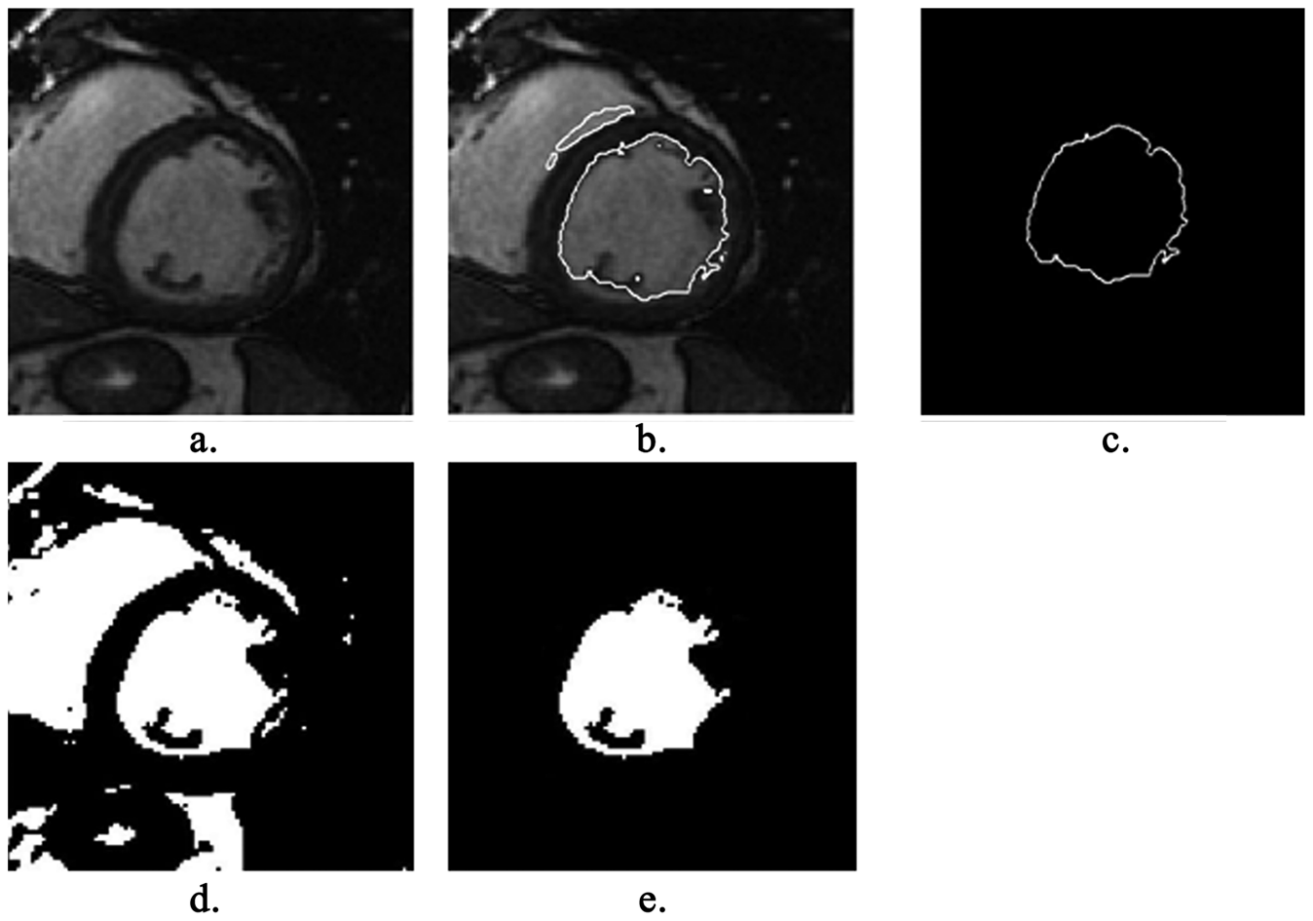
Segment a slice image using LBF model: (a) ROI of the gray image, (b) result of LBF model, (c) contour of blood pool by LBF model, (d) segment result, (e) segmented blood pool.

The energy 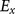 is minimized to find the object boundary 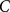 by an iterative optimization method gradient descent flow. Thus the boundary of blood pool can be obtained by the overlap of the contour area derived by LBF model and a binary mask (see Fig. 2a–2c). The binary object which has the maximal overlap area is the blood pool we are looking for. For the mid-slice image, the mask is a binary circle at the center of the image; for a non-mid-slice image, the mask is the endocardial region segmented from previous slice. Thus, the binary object derived by LBF model is used as the contour mask for LV image thresholding segmentation.

For most slices, the Otsu threshold value is suitable for locating blood pool approximately, but in some extreme cases, the blood pool is missing using Otsu thresholding method. To determine automatically the fitness of a threshold, the ROI image is segmented with different threshold values, the overlap area of detected blood pool and the contour mask by LBF model is measured. The optimal threshold corresponding to the maximal overlap value is the one we are looking for. Using above criteria, we can search a better threshold and obtain an ideal segmented blood pool (see Fig. 2d–2e).

### Step 2: Derive the boundary of endocardial

In the process of endocardial contour detection, we can find LVOT in the basal slice. In other slices, LVOT is non-existing. These two different types of slices are processed by different methods.

The basal slice with LVOT is identified by the following steps. First of all, choose the middle slice image as the start image, and process each image sequentially in the basal direction. The convex hull of the blood pool is calculated. Calculate the length of the major axis L of the ellipse that has the same normalized second central moments as the binary blood pool obtained by LBF model. Then the ratio of the current major axis L to preceding L from the previous slice image is computed. If the ratio is larger than a predefined threshold (In this work, threshold  = 1.2), basal slice with LVOT is identified [26]. Context information from the previous slice image is adopted to get endocardial contour for this type of image. Convex hull of the blood pool's boundary detected by LBF model is regarded as the initial endocardial contour for the slice image without LVOT.

**Figure 3:**
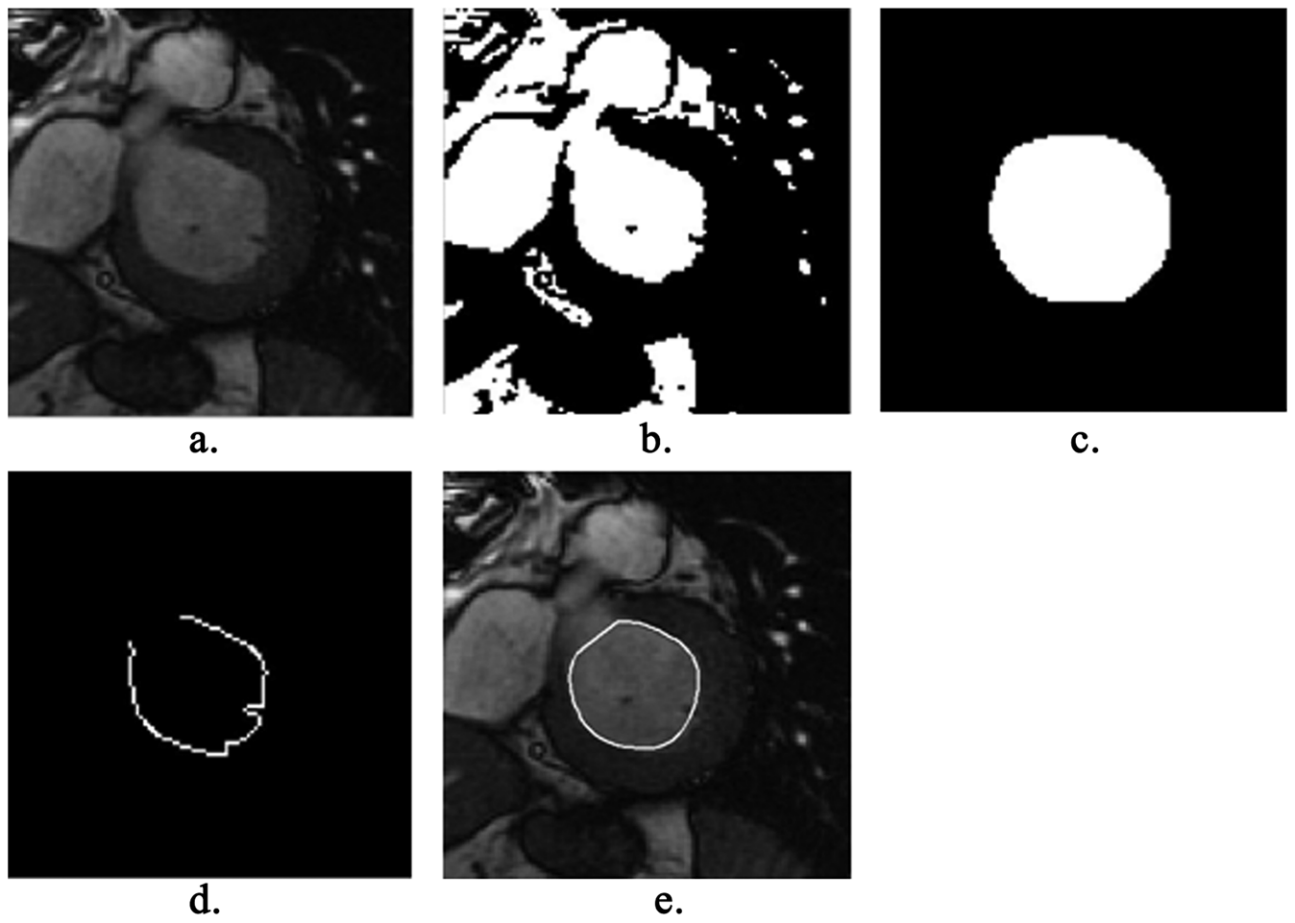
Get endocardial contour of the basal slice image with LVOT in some cases: (a) ROI of the gray image, (b) binary image of the ROI, (c) blood pool of the preceding slice image, (d) endocardial contour of the current slice image, (e) segment result of the current slice image, the solid white curve indicates the endocardial contour.

For a slice image with LVOT, we use the above LBF model to convert the gray ROI image into binary image and obtain a binary image (see Fig. 3a–3b). The dilated blood pool in the previous slice image is used as a mask to obtain the current endocardial boundary (see Fig. 3c–3d). The center point from the center of blood pool in the previous slice image is used as pole, and pole coordinate of edge points from the current endocardial boundary is derived subsequently in the form: 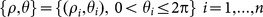; where n is the number of edge points.

For the points 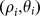, the circle fitting method is used for excluding any points for which polar radius is larger than the circle radius by a given threshold. The survived points are connected to combine an enclosed curve and convex hull for the curve is calculated as the initial endocardial contour. Bezier curve fitting [12] is used to smooth the derived contour (see Fig. 3e).

For the ROI image, the center point of obtained endocardial contour is used as the circle center, a circle is drawn with predetermined radius. The ROI image in the circle shape is transformed into a rectangular image by polar transform with the circle center point as the pole. The derived rectangular image is used to calculate the epicardial contour.

### Step 3: Calculate the epicardial contour

The Extracting of epicardial boundary is under polar system and limited to certain regions. Region restricted dynamic programming from our previous method [27] is improved for epicardial boundary.

In our previous method, region restricted dynamic programming is employed in the polar coordinate; the searching scope of the edge points for epicardial is started from the endocardial contour. Bright regions such as the right ventricle, pericardial fat and abdominal fat are excluded in the search scope for epicardial edge points; a binary mask is constructed from the context information of these bright regions. The binary mask from our previous method is improved in our schema, that is to say, the derived epicardial contour region from the preceding slice image is dilated and added to the binary mask of our previous method to construct the last binary mask which can be adopted for the following dynamic programming (DP).

In this study, we obtain an edge map of the LV image by using the non-maxima gradient suppression technique [17]. The derived edge map is used as the initial cost for DP instead of the gradient of an image which is usually applied in the classic DP [23].

The LV image is denoted as 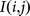 of size M×N, where M and N are the number of rows and columns, respectively. Let 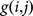 be the normalized image feature of the LV image, 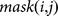 be the binary mask we have constructed to constrain the epicardial contour.
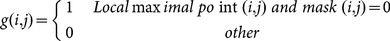
(3)


Let 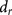 denotes the range that the point in column 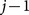 allowed to jump onto the next column 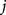, i.e., the point in column 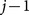 can move at most 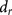 positions in either the up or down direction. For instance, if 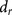  = 1, then point 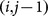 in row 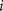 and column 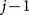 has three candidate successors (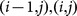, 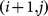) in column 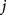.

The cost function of the point in column 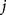 is calculated as follows:
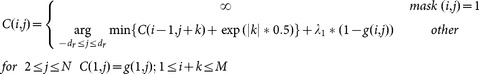
(4)where 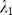 is weighting factor of the image feature 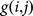. We start from the second column to the last column, the cost in current column is computed from the cost in its previous column. In this construction, small values indicate higher likely edge locations. Therefore, a cost map 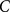 is constructed. As we know, the boundary of epicardial is enclosed, which means the last point and the first point of epicardial should be the same one. To find the position in the cost map 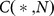 which specifies the last point of the epicardial contour, a penalty item is added to last column in the cost map, such as follows:
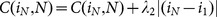
(5)where 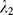 is penalty factor for the epicardial boundary; 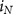 and 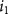 are row coordinates of the last point and the first one in epicardial contour. The position with the minimum value in the cost map 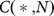 is searched, which specifies the last point of the epicardial contour. With a backward search from *N* to 1 in the columns, the complete coordinates 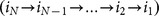 of the epicardial contour can be obtained, which are the optimal solution corresponding to the global minimum of the optimization function.

The endo- and epi-cardial contours are transformed back to the original coordinates. Then, the 1D FFT and Bezier curve fitting are used to smooth the endo- and epi-cardial contours respectively. The 1D FFT method used in this paper is defined as follows:
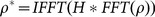
(6)where 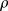 are the corresponding distances of the edge points to its center, 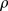 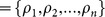. H is an ideal low pass filter, IFFT is the inverse FFT.

## Results

### 1.3 Segmentation Evaluation

We adopt the evaluation software published by the MICCAI Clinical Image Segmentation Grand Challenge Workshop to analyze our results [28]. It provides a convenient tool for researchers to test and compare their algorithms easily and objectively.

Several measures are employed in our experiments; these are percentage of good contours, average perpendicular distances, overlapping dice metric, left ventricle ejection fraction (EF) and mass (LVM) by including papillary muscles and trabeculations in the ventricular cavity.

The ground truth of LV is manually delineated by the clinical experts from the data from the same workshop. Contours generated by automatic algorithms is considered as good if the average distance from ground truth is less than 5 mm. Percentage of good contours is calculated by the number of good contours over the number of all contours of ground truth. Perpendicular distance and overlapping dice metric are calculated only in the good contours from automatic algorithms. Average perpendicular distance evaluation calculates the distance from the automatically segmented contour to the corresponding one manually drawn by an expert, averaged over all contour points. Overlapping dice metric calculates the contour areas overlapping proportion between the automatic segmentation results and the ground truth.

Clinical studies have employed different quantification methods for calculation of left ventricle volume, left ventricle mass and ejection fractions are critical parameters for cardiac diagnosis [29], [30]. LVM and EF are defined as in the following equations:
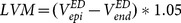
(7)

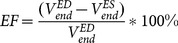
(8)


Where in the end-diastole phase, 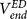 and 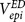 represent end- and epi-cardial volumes in the end-diastole (ED) phase respectively, while 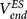 represents endocardial volume in the end-systole (ES) phase.

### 1.4 Results of 45 Cases

In this article, 45 cases have been tested in the experiment. The statistics of automatic segmentation results compared to the ground truth are listed in Table 1. Our results show that for both endo- and epi-cardial contours, percentage of good contours is about 94%, the average perpendicular distance is about 2 mm, and the overlapping dice metric is about 0.91. The average computation time of the segmentation of the proposed method is 197.99
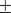
49.23 seconds per case including the time of reading DICOM data and saving contour files. The computation time is 10.96 seconds per image for the 45 cases. The computation time is tested on consumer hardware (Pentium (R) Dual-core 2.20 GHz Win XP) with a non-optimized Matlab code (Mathworks) implementation.

**Table 1:** Results on 45 studies.

		Good (%)		Distance (mm)		Overlap		EF (%)		LVM (g)	
Group	Cases	Endo	Epi	Endo	Epi	Endo	Epi	Auto	Expert	Auto	Expert
HF-I	12	*94.03*	*97.55*	*2.10*	*1.96*	*0.92*	*0.95*	*25.72*	*25.61*	*129.96*	*132.52*
HF-NI	12	*91.22*	*93.58*	*2.32*	*2.14*	*0.90*	*0.94*	*30.74*	*28.86*	*133.10*	*128.47*
HYP	12	*89.95*	*90.47*	*2.54*	*2.48*	*0.85*	*0.92*	*65.27*	*60.93*	*101.57*	*103.76*
Normal	9	*96.68*	*96.19*	*2.20*	*2.20*	*0.87*	*0.93*	*64.79*	*58.26*	*84.33*	*86.53*
All	45	*92.72*	*94.33*	*2.30*	*2.20*	*0.89*	*0.93*	*45.42*	*42.42*	*114.10*	*114.57*
Overall	45	*93.53*±*7.37*		*2.25*±*0.41*		*0.91*±*0.03*					

Distance, average perpendicular distance; EF, ejection fraction; endo, endocardial contour; epi, epicardial contour; good, percentage of good contours; HF-I, heart failure with ischemic HYP; HF-NI, heart failure without ischemic HYP; HYP, hypertrophy; LVM, left ventricle mass; overlap, overlapping dice metric.

### 1.5 Regression and Bland-Altman Analysis

**Figure 4:**
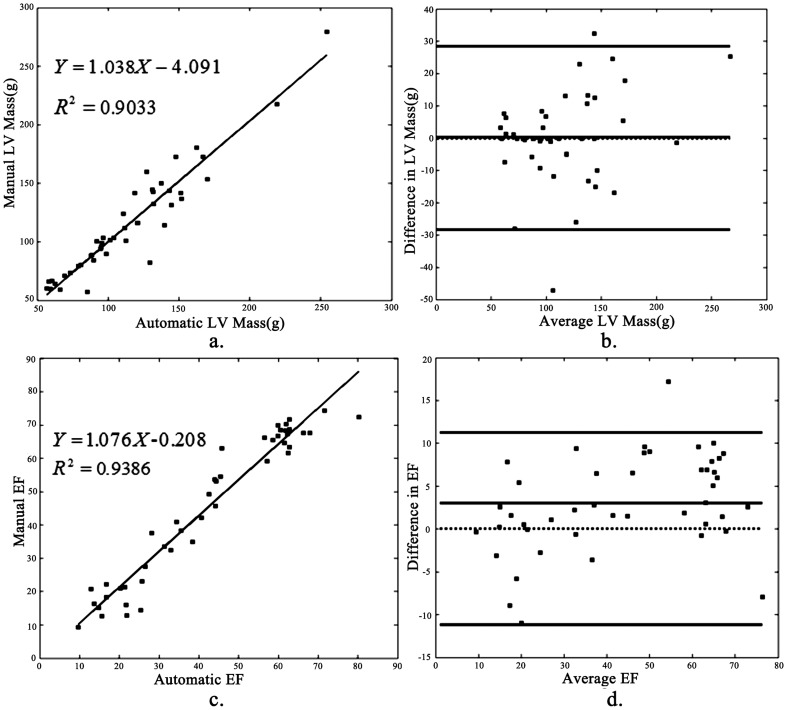
Regression curve and Bland-Altman plot for the ejection fraction (EF) and left ventricle (LV) mass: (a) linear regression for LV mass, (b) Bland-Altman plots of LV mass, (c) Linear regression for EF, (d) Bland-Altman plots of EF.

We use regression and Bland-Altman analysis to evaluate our results of 45 cases. Fig. 4 shows the regression and Bland-Altman plots for the EF and LV mass measurements. For the LV mass, the regression coefficient is very good, and the spread of the values is pretty low, the slope is 1.038, demonstrating a bias 0.27 on the Bland-Altman plot. For the EF, it can be seen that the regression coefficient is 1.076; the bias is 2.99 on the Bland-Altman plot. The coefficient of determination 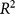 for LV mass and EF is 0.9033 and 0.9386. Therefore, the algorithm is pretty accurate at computing LV mass and the EF.

### 1.6 Comparisons

To test the performance, we compare our present approach with Huang's method [19] , our previous method [27] in the all the cases. Table 2 summarizes percentage of good contours, average perpendicular distance and overlapping dice metric from the three methods. It can be seen that percentage of good contours of our schema is larger than that of the other two methods. Consider that the average perpendicular distance and overlapping dice metric are computed in the good contours, that is to say the more percentage of good contours we get, the more slices we have calculated in the experiment. A conclusion can be drawn that our method has more accurate and robust performance than the other two methods.

**Table 2:** Compare contour accuracy in Huang's, our previous and present results.

		Mean 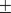 Standard Deviation		
Result		Huang's	Previous	Present
Good (%)	Endo	*79.2±19*	*91.06±9.42*	*92.72±6.86*
	Epi	*83.9±16.8*	*91.21±8.52*	*94.33±7.88*
	Overall	*81.5±18*	*91.1±8.97*	*93.5±7.37*
Distance (mm)	Endo	*2.16±0.46*	*2.24±0.4*	*2.3±0.36*
	Epi	*2.22±0.43*	*2.21±0.45*	*2.2±0.45*
	Overall	*2.19±0.45*	*2.23±0.43*	*2.25±0.41*
Overlap	Endo	*0.89±0.04*	*0.89±0.03*	*0.89±0.04*
	Epi	*0.93±0.02*	*0.94±0.02*	*0.93±0.02*
	Overall	*0.91±0.03*	*0.92±0.03*	*0.91±0.03*

We compare percentage of good contours of 15 cases from the second dataset among Lu's [18], Wijnhout's [31] and our results, as shown in Table 3. Overall percentage of good contours from Lu's and Wijnhout's is 76.78
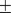
21.74 (%) and 90.37
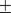
21.62 (%), respectively; it is 93.75
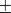
6.26 (%) for our method. It shows that our method has better performance in percentage of good contours than the two other algorithms.

**Table 3:** Compare percentage of good contours among Lu's, Wijnhout's and our results in the second dataset.

	Lu's (%)		Wijnhout's (%)		Our (%)	
patient_id	Endo	Epi	Endo	Epi	Endo	Epi
SC-HF-I-05	*83.33*	*88.89*	*100*	*100*	*100.00*	*100.00*
SC-HF-I-06	*95.45*	*100*	*100*	*92*	*95.45*	*100.00*
SC-HF-I-07	*87.50*	*100*	*75*	*100*	*87.50*	*100.00*
SC-HF-I-08	*50*	*63.64*	*100*	*100*	*90.91*	*100.00*
SC-HF-NI-07	*87.50*	*91.67*	*92*	*100*	*91.67*	*83.33*
SC-HF-NI-11	100	80	100	90	100.00	100.00
SC-HF-NI-31	*63.16*	*70*	*84*	*100*	*94.44*	*100.00*
SC-HF-NI-33	*83.33*	*90*	*89*	*90*	*88.89*	*100.00*
SC-HYP-06	*76.92*	*57.14*	*85*	*100*	*84.62*	*100.00*
SC-HYP-07	*50*	*87.50*	*69*	*100*	*81.25*	*87.50*
SC-HYP-08	*42.11*	*80*	*68*	*90*	*78.95*	*90.00*
SC-HYP-37	*46.15*	*57.14*	*85*	*86*	*84.62*	*100.00*
SC-N-05	*53.33*	*75*	*80*	*100*	*93.33*	*100.00*
SC-N-06	*84.62*	*85.71*	*92*	*86*	*100.00*	*85.71*
SC-N-07	*83.33*	*90*	*78*	*80*	*94.44*	*100.00*
All	*72.45±18.86*	*81.11±13.47*	*86.47±10.65*	*94.27±6.65*	*91.07±6.49*	*96.44±6.04*
Overall	*76.78±21.74*		*90.37±21.62*		*93.75±6.26*	

**Figure 5:**
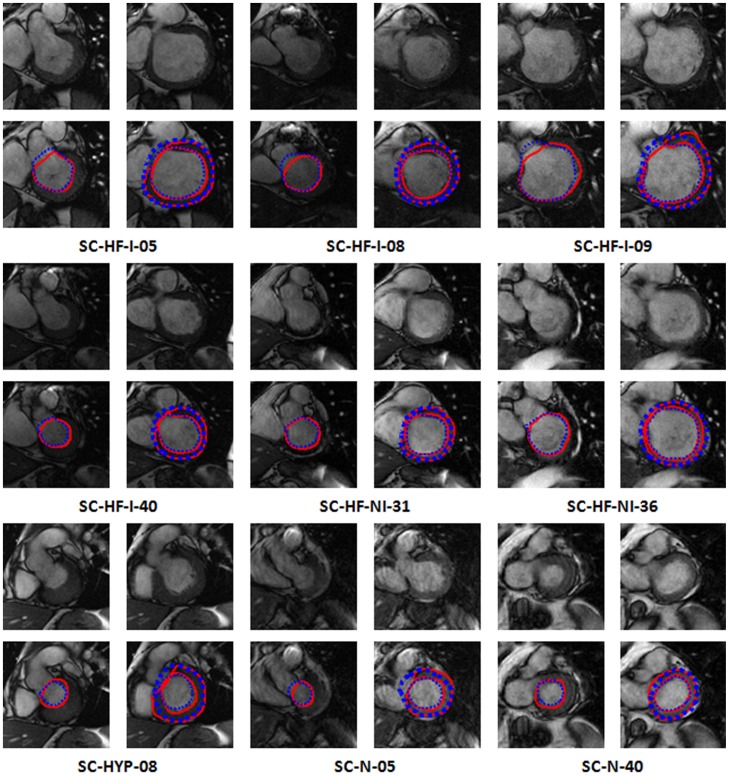
Some segmentation outputs from 9 studies with LVOT by our method; for each case, four images are shown. The left ones are the gray image and its segmentation results from the ES phase; the right ones are derived from the ED phase. The names below the image data are from the data source. The dashed blue curves indicate our contours; while the solid red ones are the ground truth. The epicardial contours are not drawn by experts in the ES phase (cropped for better viewing).

We reveal some segmentation outputs from 9 studies with LVOT by our method in Fig. 5, the names below the image data are from the data source [28]. Segmentation results from our method and clinical experts are in different styles. The contours from experts are solid red curves; while the outputs from ours are dashed blue ones. For each case, four images are shown. The left ones are the gray image and its segmentation result from the ES phase; while the right ones are derived from the ED phase. The ground truth for the epicardial contour of the LV in ES phase is omitted by clinical experts, because it is not used when computing ejection fraction and left ventricle mass by the published evaluation software.

**Figure 6:**
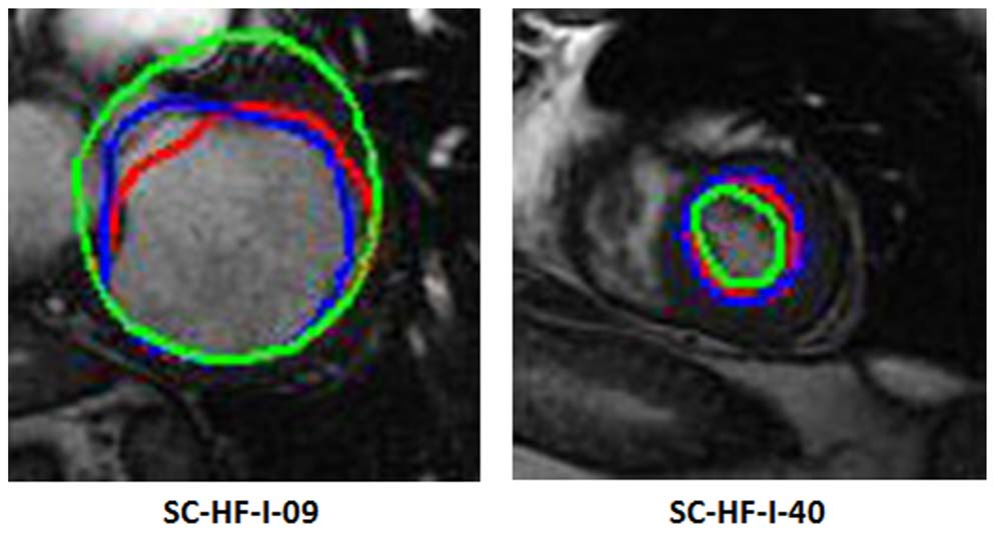
Endocardial contours of two slice images from case SC-HF-I-09 and SC-HF-I-40. The blue curves are obtained using our present algorithm, whereas the green ones are from our previous method. The red curves are the ground truth.

To demonstrate the advantages of our proposed method, we compare the derived endocardial contours from 2 studies (SC-HF-I-09, SC-HF-I-40) obtained from two methods in Fig. 6. For each case, two resulting images are shown. The blue curves come from our present method, whereas the green ones are from our previous method. The red curves are the ground truth. The comparison results indicated that the endocardial outlines segmented by our proposed method match experts' outlines more closely, compared to our previous method.

**Figure 7:**
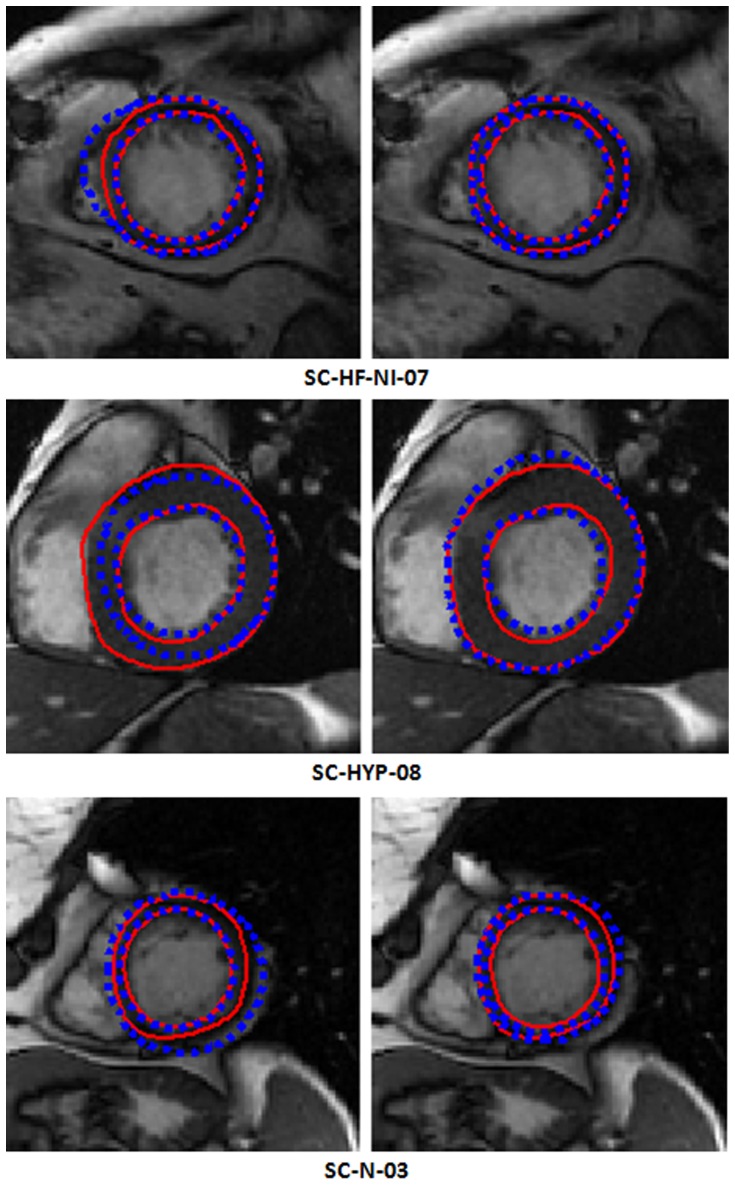
Endo- and epi-cardial contours from 3 cases derived from two methods. For each case, two images are shown. The left one is the segmentation result from our previous method; while the right one is derived from our present algorithm. The ground truth from experts is drawn in solid red curve; whereas the dashed blue one represents the results of our previous or present method.

Furthermore, we display segmentation outputs for both endo- and epi-cardial contours from 6 studies obtained by two approaches in Fig. 7. For each case, two resulting images are shown. The left one is the outcome from our previous method and the right one comes from our present algorithm. The dashed blue ones represent the results of our previous method or the present algorithm; while the solid red curves are the ground truth. It can be seen that the schema proposed by us does better performance than our previous method.

## Discussion

In this article, an automatic segmentation schema is developed for left ventricle on short-axis cardiac in multislice MRI. A series of image processing techniques are adopted in the schema, such as active contour model, optimal thresholding, non-maxima gradient suppression, and region constrained dynamic programming. The experiments show that the performance of our method is very promising.

In LV segmentation, a very important step is to locate the LV blood pool which is often solved by region based technique, such Gaussian-mixture model and Otsu thresholding. The performance of Gaussian-mixture based segmentation algorithms clearly depends on how well the histogram of an image approximates a Gaussian mixture and the accuracy of the estimates of the model parameters. If the histograms of real LV images do not approximate a Gaussian mixture, it may produce wrong segmentation results. The Otsu method [20] selects the optimal threshold by maximizing distinguishes of the object and the background class in gray level. If the histogram of an image has the bimodal distribution and possesses a deep and sharp valley between two peaks, the Otsu method exhibits the relatively good performance. However, if the variances of the object and the background gray levels are large compared with the mean difference (that is, the difference of the average gray levels of the object and the background), the sharp valley of the gray level histogram is degraded. Then the possibly incorrect threshold determined by the Otsu method results in the segmentation error. Recently, Xu et al. [32] revealed that the Otsu method could be biased from the optimal threshold. They proved that the bias is due to differences in class variances or class probabilities, and the obtained threshold is biased towards the component with larger class variance or larger class probability.

This study designs a simple but effective measure to solve the above problem. At first, local binary fitting model which combines edge and region information is used to locate blood pool of the LV image. Then the optimal threshold is obtained for LV segmentation. For the slice image with LVOT, the endocardial contour is extracted using context information from the previous slice image.

For epicardial boundary extraction, we improve dynamic programming with some techniques, such as non-maxima gradient suppression, region constrained technique and context information constrain from the epicardial contour of the preceding slice image. Experimental results for the epicardial contour show that the above techniques are very effective.

In our automatic LV segmentation in short-axis cardiac MRI, a slice-by-slice segmentation of LV image starts from mid-slice and extends to two-end slices, and the current slice center and the relevant masks from the obtained contours is used for next slice. The use of prior knowledge about spatial relationships between slice images makes our method accurate and robust.

Segmentation performance of our approach and other methods are compared by using the measures of percentage of good contours, average perpendicular distance, and overlapping dice metric. Clinic parameters (EF and LVM) are studied using regression and Bland-Altman analysis. The results indicate that our proposed method has more accurate and robust performance.

Although our schema is robust enough to cope with most studies, some extreme cases are still a challenge: 1) overlap between the intensity distributions within the cardiac regions in some slices; 2) the small blood pool at the apex is difficult to detect correctly. How to further improve accuracy and time efficiency of LV segmentation will be studied in the future.
